# Evidence of unidirectional gene flow in a fragmented population of *Salmo trutta* L.

**DOI:** 10.1038/s41598-021-02975-9

**Published:** 2021-12-03

**Authors:** Rafał Bernaś, Anna Wąs-Barcz, Mariann Árnyasi, Piotr Dębowski, Grzegorz Radtke, Anita Poćwierz-Kotus, Patrick Berrebi

**Affiliations:** 1 Department of Migratory Fishes, Inland Fisheries Institute, Rutki 49, 83-330 Zukowo, Poland; 2 Department of Fisheries Resources, National Marine Fisheries Research Institute, Kołłątaja 1, 81-332 Gdynia, Poland; 3 Department of Animal and Aquacultural Sciences, Faculty of Biosciences, Centre for Integrative Genetics (CIGENE), Norwegian University of Life Sciences, Ås, Norway; 4 Institute of Oceanology, Polish Academy of Sciences, Powstańców Warszawy 55, 81-712 Sopot, Poland; 5 Genome-Recherche and Diagnostic, 697 avenue de Lunel, 34400 Saint-Just, France

**Keywords:** Fisheries, Genetic variation, Ichthyology

## Abstract

Selection, genetic drift, and gene flow affect genetic variation within populations and genetic differences among populations. Both drift and selection tend to decrease variation within populations and increase differences among populations, whereas gene flow increases variation within populations but leads to populations being related. In brown trout (*Salmo trutta* L.), the most important factor in population fragmentation is disrupted river-segment connectivity. The main goal of the study was to use genetic analysis to estimate the level of gene flow among resident and migratory brown trout in potential hybridization areas located downstream of impassable barriers in one river basin in the southern Baltic Sea region. First, spawning redds were counted in the upper river basin downstream of impassable barriers. Next, samples were collected from juveniles in spawning areas located downstream of barriers and from adults downstream and upstream of barriers. Subsequently, genetic analysis was performed using a panel of 13 microsatellite loci and the *Salmo trutta* 5 K SNP microarray. The genetic differentiation estimated between the resident form sampled upstream of the barriers and the anadromous specimens downstream of the barriers was high and significant. Analysis revealed that gene flow occurred between the two forms in the hybridization zone investigated and that isolated resident specimens shared spawning grounds with sea trout downstream of the barriers. The brown trout population from the river system investigated was slightly, internally diversified in the area accessible to migration. Simultaneously, the isolated part of the population was very different from that in the rest of the basin. The spawning areas of the anadromous form located downstream of the barriers were in a hybridization zone and gene flow was confirmed to be unidirectional. Although they constituted a small percentage, the genotypes typical upstream of the barriers were admixed downstream of them. The lack of genotypes noted upstream of the barriers among adult anadromous individuals might indicate that migrants of upstream origin and hybrids preferred residency.

## Introduction

Brown trout (*Salmo trutta* L.) is a Palearctic salmonid species naturally distributed in Europe from the White Sea to North Africa and from the UK to western Asia^1^. It is a highly polymorphic teleost fish with several life strategies. Anadromous trout, referred to as sea trout, migrate from natal rivers or streams to the sea, where they feed until reaching sexual maturity, and subsequently return to their natal rivers to spawn. In contrast, resident trout spend their entire lives in rivers or streams and often spawn in the same area in smaller tributaries upstream^2^. Brown trout populations are of great economic importance as they are a significant component of fishery resources, and they play an important role in angling tourism in many European countries.

In Poland, there are about 25 rivers in which sea trout occur. Resident brown trout populations inhabit parts of Polish river catchments, mainly in northern and southern Poland, which is generally consistent with the historical range of the migratory form before river-sea connectivity was destroyed by human activity. All river populations are considered to be admixed with domestic strains after more than 40 years of stocking^3^ with several domestic lineages^4^.

The phenomenon of migratory and resident individuals coexisting in the same population is a common expression of life history plasticity in fishes^5^. Among individuals, the decision to migrate is controlled by both genetic and environmental factors^6–8^. Despite clear differences in life cycles, the extent of direct and indirect biological interactions that are in sympatry through competition for food or space and the extent of reproductive isolation between these two forms are still disputed. The two forms can use the same locations for spawning during overlapping periods and are morphologically identical at the juvenile stage^9^. In general, several laboratory techniques have been used to identify resident and anadromous fishes, including carotenoid pigment profiling^10^, strontium content of scales and bony tissues^11,12^, stable isotope ratio analysis^13^, and microsatellite analysis by, e.g., parentage analysis^14^, or a combination of techniques, which can lead to a significant increase in the proportion of fish for which life history can be reliably determined^15^. However, with the exception of genetic studies and combined studies, these methods are used only to identify adult fish that have spent time in the marine environment or very recently hatched juvenile fry spawned from a sea trout mother. Studies from Normandy based on stable isotope ratios revealed gene flow between resident and anadromous brown trout forms^16,17^. In the Kerguelen Islands, an experiment showed that an Atlantic hatchery strain released in a troutless river produced both resident and anadromous forms^18^ demonstrating that, fundamentally, fry can manifest resident or migratory lifestyles.

It is known that an impassable barrier can induce genetic differentiation between upstream and downstream fish populations^19^. Thus far, issues related to the organization of the brown trout population in the river basin analyzed have not been studied specifically in terms of differences resulting from the presence of impassable barriers. The contributions of migrants from the upper part of the catchment isolated by an impassable barrier, if indeed it is impassable, remain unknown. Earlier studies^20^ suggested that some form of gene flow could occur between populations isolated by barriers and those located downstream. The hypothesis that unidirectional, downstream migration from isolated areas mitigates discontinuity in correlation with genetic and geographic distance will be tested.

The main objectives of this work were to estimate the level of gene flow between resident and migratory brown trout using genetic analysis in potential hybridization areas located downstream of impassable barriers in the Parsęta River. Microsatellite and SNP microarray genotyping results were also compared to check the complementarity of markers and the efficiency of estimations.

## Material and methods

### Study area

**Figure 1:**
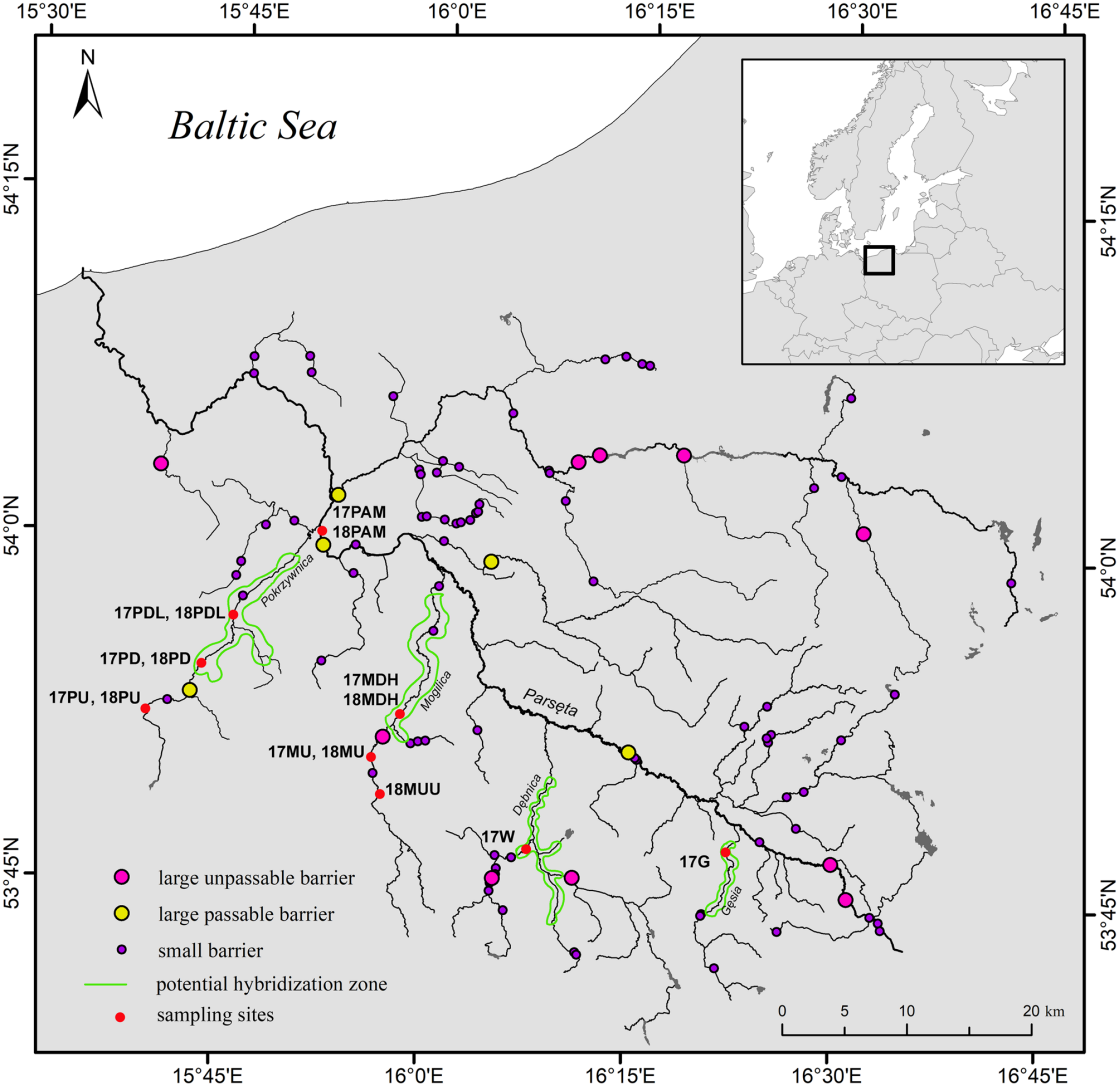
Parsęta River basin with migration barriers, potential hybridization zones and sampling sites (created by author in ArcMap 10.7.1).

The Parsęta River is the largest Pomeranian river with a length of 143 km and a catchment area of 3084 km^2^ (Fig. 1). The average flow is about 30 m^3^ s^–1^. The Parsęta and most of its main tributaries flow through forested valleys with meadows and through areas of fields and forest. The river passes some small towns on its way to the sea, and it enters the Baltic proper at the harbor town of Kołobrzeg (ICES rectangle no. 25). The majority of the Parsęta River and its main tributaries remain largely natural^21^. The fish fauna is represented by approximately 20 species with *Salmo trutta* L. and *Cottus gobio* L. being dominant^22^.

There are about 100 hydrotechnical barriers located in the Parsęta basin. Among the studied tributaries, the Pokrzywnica River is fully free-flowing up to 18 km and almost along its entire length at higher water levels. The Mogilica River is unaffected by any barriers up to 27 km, where there is a smagll hydroelectric plant on the site of an old mill dating from 1850, and another old mill is located about 4 km above this point. The Dębnica River has 21 km that are free for migration, but its tributary the Wogra is only free for 8 km (Fig. 1). The anadromous sea trout form can migrate a distance of 144 km along the Parsęta River (Fig. 1). The locations of the impassable and passable barriers are presented in Fig. 1. Several hundred sea trout are caught annually by electrofishing in the middle part of the river basin for use in artificial spawning; catches are made by fishers authorized to exploit these river stocks. The number of sea trout entering the Parsęta River annually is up to several thousand^3^. Sea trout spawning is monitored by counting redds in the tributaries and the upper part of the main river. Female trout excavate nests in gravel substrates, deposit eggs that are fertilized externally by one or more males, then quickly cover these with gravel and begin to dig other nests. A contiguous series of these nests is called a redd^23^. In many salmonid rivers, long time-series of redd count data is sometimes available for salmonid populations^24^. In 2017 and 2018, several potential hybridization zones located downstream of barriers were selected based on redd counts at sites where hybridization between isolated and potentially differentiated residents upstream of barriers (or their descendants) and anadromous forms was anticipated (Fig. 1).

### Sample collection and DNA extraction

Sampling began in summer 2017 when 260 juveniles aged 0 + were collected at 7 locations and 65 anadromous adults were collected in the middle part of the main river (Fig. 1, Table 1). The mean size of anadromous spawners was 60.5 cm (53.5–86.0 cm). In 2018, 204 juveniles 0 + and 50 anadromous adults were collected from 6 locations (Fig. 1, Table 1). The mean size of anadromous spawners from 2018 was 61.5 cm (51.5 – 81.0 cm). In total, fin clip samples (approximately 2–5 mm^2^) were collected from 579 individuals. Genomic DNA was extracted from fin tissue preserved in 96% ethanol with Genomic Mini Kits (A&A Biotechnology) and diluted to a concentration of 30–100 ng/µl. DNA solutions were measured in a Qubit fluorimeter (Thermo Fisher) and normalized to a concentration of 50 ng/µl to perform genotyping via an SNP microarray. Sampling details are presented in Table 1. The key to sample abbreviation is as follows: the numbers indicate the sampling year, the first letter indicates the name of the river, the second—a special feature (U = upstream, D = downstream), the third—additional details (H = hybrid zone, L = lower, M = main, U = upstream).

**Table 1:**
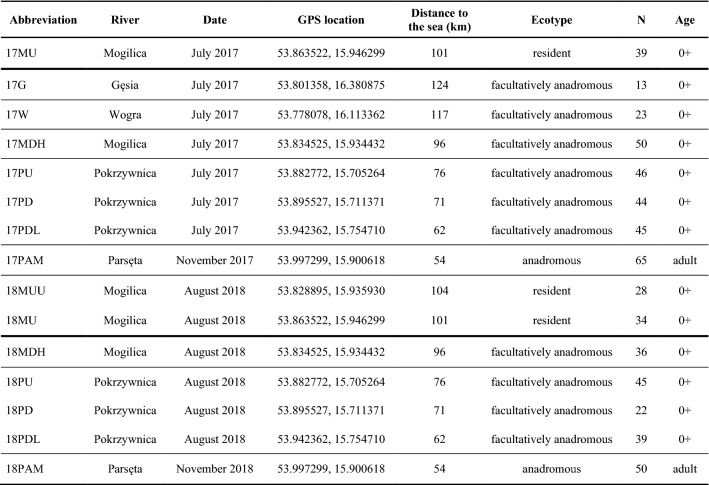
Details of brown trout sampling in the Parsęta basin in 2017–2018.

Sites are ordered according to sampling year, blocked tributaries (bold black line), and distance to the Baltic Sea. Black lines indicate separate rivers.

### Microsatellite analysis

A set of 13 fluorescently labelled polymorphic microsatellite loci—*OneU9*, *Strutta58P*, *Ssosl438*, *Ssosl311*, *Str15INRA*, *Str543INRA*, *Str60INRA*, *Str73INRA*, *Ssosl417*, *Str85INRA*, *Ssa85*, *Bs131*, *Ssa407*^25–32^—were amplified in a single multiplex PCR reaction using Qiagen Multiplex PCR Kits (Qiagen, Germany). The 7 µl multiplex PCR reaction was performed with about 100 ng of template DNA, multiplex PCR master mix, and 0.2–0.6 µM of each primer. Amplifications were carried out in a TProfessional Basic Gradient thermal cycler (Biometra) with an initial heat of 95 °C for 5 min followed by 38 cycles of denaturation at 94 °C for 30 s, annealing at 55 °C for 90 s, and extension at 72 °C for 60 s. The PCR was terminated after 30 min and the final extension was at 60 °C. PCR products were genotyped in single capillary electrophoresis on an ABI Prism 3130xl genetic analyzer (Applied Biosystems) along with GeneScan 600LIZ size standard (Applied Biosystems). DNA fragments were estimated using a Peak Scanner v1.2 (Applied Biosystems).

### SNP genotyping and SNP validation

Part of the samples collected in 2017 and 2018 were genotyped with a brown trout (*S. trutta*) Illumina iSelect SNP microarray that was custom developed at the Centre for Integrative Genetics (CIGENE), Norway^33,34^. In total, 144 trout were genotyped. The array was based on assays for 5509 SNP loci. The results were manually inspected using GenomeStudio^®^ (version 2011.1, Illumina Inc., San Diego, CA, USA), and 1538 SNPs were excluded for one or more of the following reasons: (1) cluster patterns suggested multi-site or paralogous site variants; (2) unknown nearby variant (null allele); (3) a SNP was monomorphic or mitochondrial. The results were transferred to a spreadsheet and the acceptable threshold of missing data across samples was determined at 5%, which led to the removal of 14 more SNPs. Another 75 loci with minor allele frequencies (MAF) of less than 0.01 were also discarded. After filtering, the data from the remaining a set of 3882 polymorphic SNPs were analyzed.

### Statistical analysis

The following estimates were made for the calculations based on microsatellite DNA. Observed and expected heterozygosity and the mean number of alleles (total number of alleles at all loci divided by the number of loci) were calculated using Arlequin 3.5.2.2^35^. Population specific F_IS_, pairwise weighted F_ST_ values over all loci based on the number of different alleles, and Nei’s genetic distances were also determined with this software. Departures from the Hardy–Weinberg Equilibrium (HWE) were detected with Chi-square tests in GenAlex 6.5^36^. HPRARE was used to calculate allelic richness (which allows comparison of allele numbers without the bias associated with different sample sizes) and the richness of private alleles (alleles limited in a single population)^37^. Overall, the F-statistic (F_ST_, F_IT_, F_IS_) was estimated by analyzing molecular variance (AMOVA) implemented in Arlequin 3.5.2.2. STRUCTURE 2.3.4 was applied to detect genetic structure and gene flow^38^. The Evanno method (∆K) was chosen^39^ to infer the best number of clusters (K) based on the rate of change in log probability among consecutive K values. Five iterations of each K were performed with 100,000 burn-ins and 200,000 Markov Chain Monte Carlo (MCMC) repetitions. Then the Clumpak program identified the optimal alignment of inferred clusters across different values of K^40^. Next, STRUCTURE results were used to detect hybrids. The membership coefficients (*q*) obtained for optimal K were averaged from five independent runs. When the value of *q* was higher than 0.8 for any cluster, the fish were categorized as clade members. When *q* values were estimated between 0.8 and 0.2, which are conservative thresholds, the fish were categorized as hybrids. Additionally, genetic heterogeneity and hybrid detection was tested with the pairwise assignment tests in GenAlex 6.5^36^. A second hybrid detection method was used to validate and authenticate the results from the STRUCTURE algorithm. Individuals for which the values of the difference of the assessment logarithms of likelihood were closest to zero and were up to 20% of this value were assumed to be potential hybrids. Individuals with higher values of belonging to genotypes upstream of a barrier (MD) were treated as F0 migrants.

The same scheme was performed for calculations based on SNPs except for allelic richness and private allele estimations. Instead, the number of polymorphic loci and the mean number of alleles are shown, both of which were calculated in Arlequin 3.5.2.2. The sequential Bonferroni correction^41^ was also applied to detect deviations from HWE.

### Ethical approval

All methods were carried out in accordance with relevant guidelines and regulation. The study complies with the current laws of the Republic of Poland. All applicable international, national, and institutional guidelines for the care and use of animals were followed (Certificate no. 3798/2016 for Rafał Bernaś by the Polish Laboratory Animal Science Association). Field protocols for the capture, handling, and release of fish were approved by the Department of Environmental Protection, Marshal’s Office of the West Pomeranian Voivodeship (Certificate no. WRiR-I.7143.29.2017.TM) and the Water Management Department in the Union of Towns and Communes of the Parsęta River Basin (Certificate no. ZDPII.04.611.15.2017.AM).

## Results

### Microsatellites genetic polymorphism and diversity

**Figure 2:**
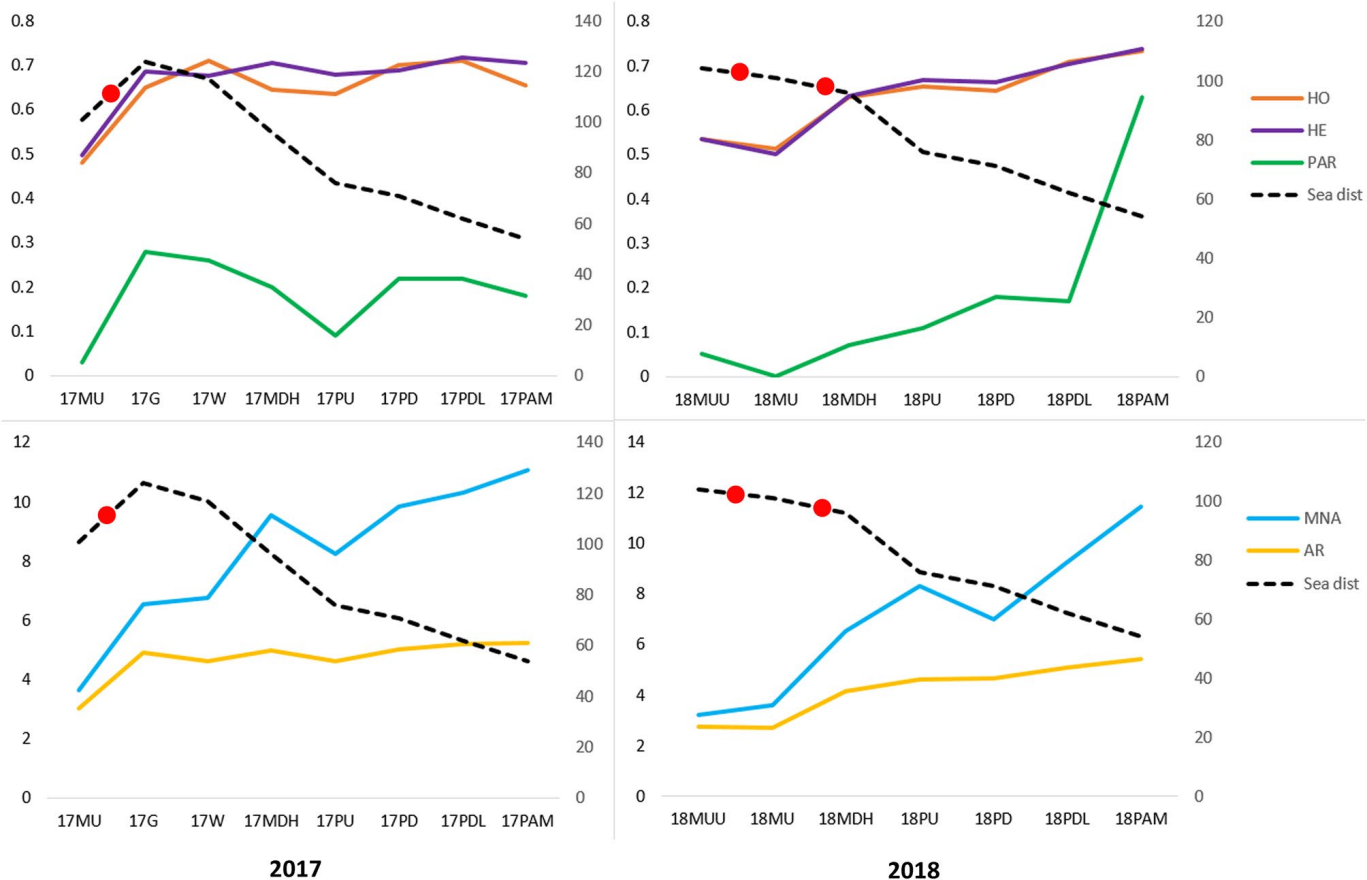
Allelic patterns and heterozygosity in brown trout from the Parsęta River basin sampled in 2017 (left) and 2018 (right). *MNA* mean allele number in the population, *HO* observed heterozygosity, *HE* expected heterozygosity, *AR* allelic richness *PAR* private allele richness, *Sea dist* distance to the sea (km). Red dots show location of impassable barriers.

The mean number of alleles in individuals from the stocks investigated ranged between 3.23 and 11.46 (Table 2; Fig. 2). The lowest values were found for trout sampled upstream of barriers, especially above the second mill (18MUU). In general, the mean number of alleles decreased in tributaries according to the distance from the main river. Observed heterozygosity was also lowest in trout upstream of the barriers and was the highest in adult anadromous spawners, similar to allelic richness and private alleles (Table 2; Fig. 2). The highest number of loci with significant departures from the HWE (Chi^2^ p < 0.05) was found in specimens that originated from the upper part of the Pokrzywnica River (17PU and 18PU). Population-specific F_IS_ values were insignificant (p < 0.05) in all stocks.

**Table 2:** Basic statistics of brown trout collected in the Parsęta basin in 2017 and 2018.

Abbreviation	N	MNA	H_O_	H_E_	A_R_	P_AR_	DHWE	F_IS_
17MU	44	3.62	0.481	0.496	3.01	0.03	1	− 0.01
17G	13	6.54	0.649	0.686	4.92	0.28	1	0.05
17W	23	6.77	0.709	0.675	4.62	0.26	1	− 0.05
17MDH	50	9.54	0.645	0.706	4.98	0.2	2	0.09
17PU	46	8.23	0.634	0.679	4.61	0.09	4	0.07
17PD	44	9.85	0.701	0.687	5.02	0.22	1	− 0.02
17PDL	45	10.31	0.711	0.716	5.21	0.22	2	0.01
17PAM	65	11.08	0.653	0.705	5.22	0.18	3	0.07
18MUU	28	3.23	0.536	0.534	2.74	0.05	1	0
18MU	34	3.62	0.514	0.502	2.71	0	1	− 0.02
18MDH	36	6.54	0.63	0.632	4.17	0.07	0	0
18PU	45	8.31	0.653	0.668	4.63	0.11	3	0.02
18PD	22	7	0.643	0.662	4.65	0.18	0	0.03
18PDL	39	9.23	0.71	0.705	5.09	0.17	1	− 0.01
18PAM	50	11.46	0.734	0.737	5.44	0.63	1	0

*N* number of fish, *MNA* mean allele number in the population, *H*_*O*_ observed heterozygosity, *H*_*E*_ expected heterozygosity, *A*_*R*_ allelic richness, *P*_*AR*_ private allele richness, *DHWE* number of loci with deviations from HWE, *F*_*IS*_ stock-specific inbreeding coefficient.

The highest F_ST_ values for pairwise difference were observed between populations upstream of the barriers in the Mogilica River (17MU, 18MUU and 18MU) and all other populations across catchment (0.098 < F_ST_ < 0.216, Table 3). The level of this differentiation was very high and was the highest for the pair of 18MUU vs. 17G (F_ST_ = 0.216). Samples collected upstream of the first barrier on this river (17MU and 18MU) were relatively closely related to samples collected at the uppermost site (18MUU) upstream of the second barrier (0.040 < F_ST_ < 0.053). Values between all other populations were largely lower (0.005 < F_ST_ < 0.067). The lowest pairwise difference was detected for samples collected in the Pokrzywnica River pair 17PD vs. 18PDL (F_ST_ = 0.05) and between adult anadromous spawners (17PAM) and samples from the Pokrzywnica River (17PD) (0.007, Table 3). Overall F_ST_ obtained by AMOVA for all pairs of loci was 0.067 and was significant. The highest percentage of variation was detected within individuals at 90.94%. Overall, F_IS_ and F_IT_ were 0.02 and 0.09, respectively, and were significant (p < 0.05).

**Table 3:** Genetic diversity indices for brown trout samples collected in the Parsęta basin in 2017 and 2018.

	17MU	17G	17W	17MDH	17PU	17PD	17PDL	17PAM	18MUU	18MU	18MDH	18PU	18PD	18PDL	18TPAM
17MU	**6.537**	1.732	1.946	1.012	1.605	1.446	1.447	1.439	0.366	0.263	1.46	1.573	1.299	1.44	1.528
17G	0.198	**8.788**	0.503	0.304	0.23	0.323	0.258	0.172	1.94	1.54	0.589	0.396	0.55	0.348	0.528
17W	0.209	0.054	**8.781**	0.562	0.532	0.491	0.349	0.511	1.967	1.763	0.496	0.567	0.517	0.454	0.625
17MDH	0.111	0.032	0.058	**9.184**	0.234	0.197	0.166	0.079	1.252	0.883	0.327	0.34	0.248	0.181	0.201
17PU	0.17	0.025	0.057	0.025	**8.833**	0.198	0.237	0.169	1.804	1.466	0.443	0.105	0.359	0.218	0.369
17PD	0.156	0.035	0.052	0.021	0.022	**8.933**	0.09	0.06	1.633	1.23	0.325	0.191	0.099	0.041	0.144
17PDL	0.152	0.027	0.037	0.018	0.025	0.01	**9.301**	0.06	1.518	1.264	0.378	0.306	0.162	0.14	0.178
17PAM	0.148	0.018	0.053	0.009	0.018	0.007	0.006	**9.166**	1.677	1.278	0.373	0.263	0.197	0.076	0.12
18MUU	0.053	0.216	0.209	0.132	0.185	0.17	0.155	0.166	**6.403**	0.271	1.648	1.776	1.575	1.626	1.712
18MU	0.039	0.18	0.192	0.098	0.157	0.134	0.134	0.133	0.04	**6.531**	1.154	1.474	1.115	1.276	1.32
18MDH	0.165	0.067	0.056	0.036	0.049	0.036	0.041	0.04	0.181	0.135	**8.211**	0.387	0.266	0.336	0.418
18PU	0.169	0.044	0.061	0.037	0.012	0.021	0.033	0.028	0.185	0.159	0.044	**8.683**	0.337	0.174	0.394
18PD	0.152	0.06	0.056	0.026	0.039	0.011	0.017	0.021	0.177	0.133	0.031	0.037	**8.609**	0.148	0.207
18PDL	0.154	0.036	0.048	0.019	0.024	0.005	0.015	0.008	0.168	0.138	0.037	0.019	0.016	**9.165**	0.147
18TPAM	0.155	0.052	0.062	0.021	0.038	0.015	0.019	0.013	0.168	0.136	0.044	0.041	0.021	0.015	**9.578**

F_ST_ values for pairwise comparisons, which were all significant (p = 0.05), are below the diagonal (bold value); the average numbers of within-stock pairwise differences are on the diagonal (bold value); Nei’s genetic distances D_A_ are above the diagonal (bold value).

### Microsatellite genetic structure

**Figure 3:**
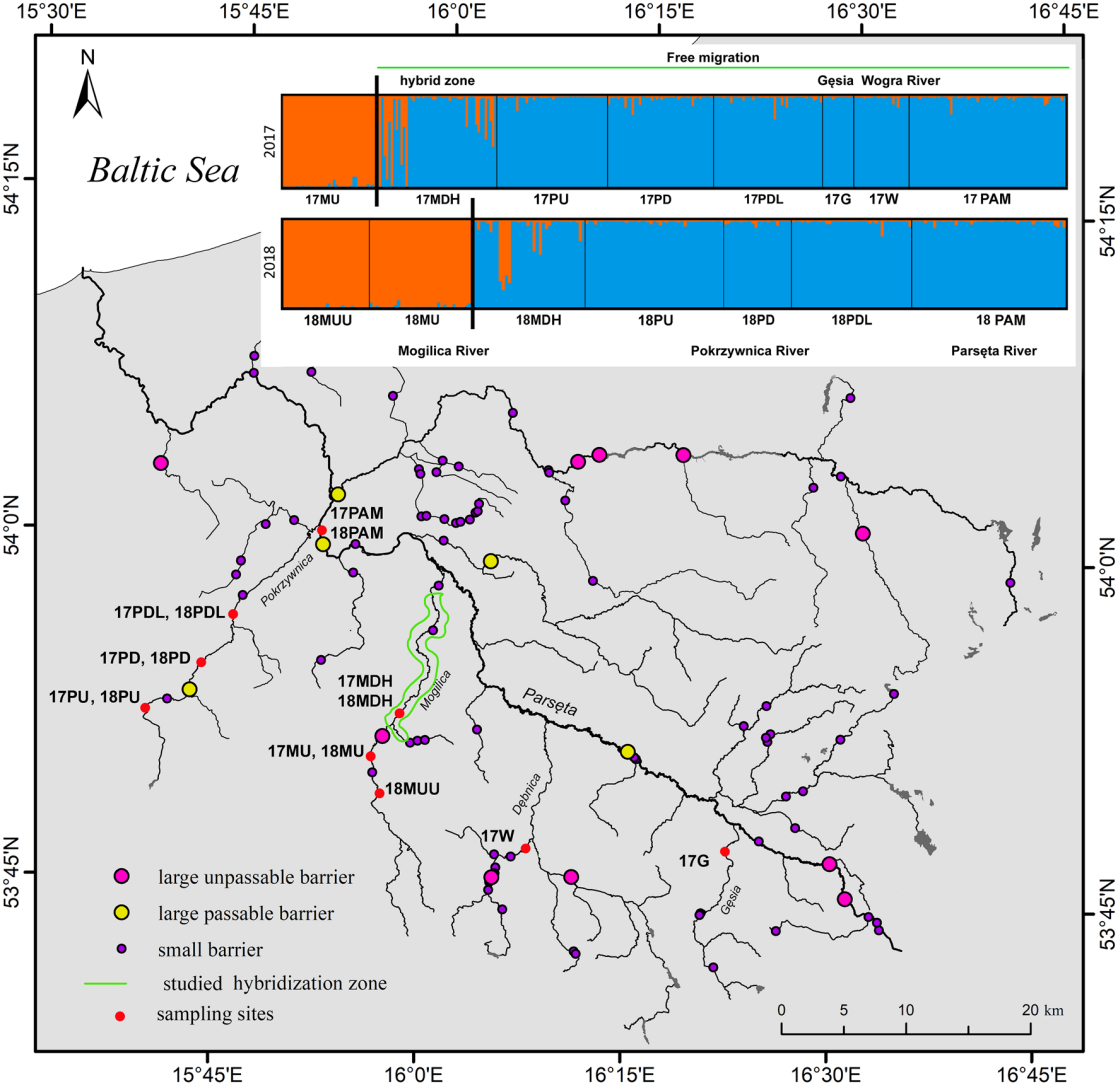
Clustering of brown trout collected in the Parsęta basin in 2017 (upper bar) and 2018 (lower bar), K = 2. Each individual is represented by a column divided into K shades with each shade representing membership of a particular cluster (created by author in ArcMap 10.7.1).

To understand the spatial distribution in the brown trout population from the Parsęta River, the genetic structure of all samples collected in 2017 and 2018 was analyzed. The Bayesian estimation of genetic structure and individual membership indicated that the maximum value of ΔK was K = 2 in both 2017 (ΔK = 125.4) (Supplementary Fig. S1) and in 2018 (ΔK = 493.65) (Supplementary Fig. S2). Brown trout from a tributary blocked by barriers clustered separately (17MU, 18MU, and 18MUU). All other locations were in one cluster that corresponded to the part of the river basin that was open to migration (Fig. 3). In both years, evidence of mixed genotypes was detected in samples collected from spawning areas downstream of impassable barriers in the Mogilica River (17MDH and 18MDH).

### Hybrid detection based on microsatellites

**Figure 4:**
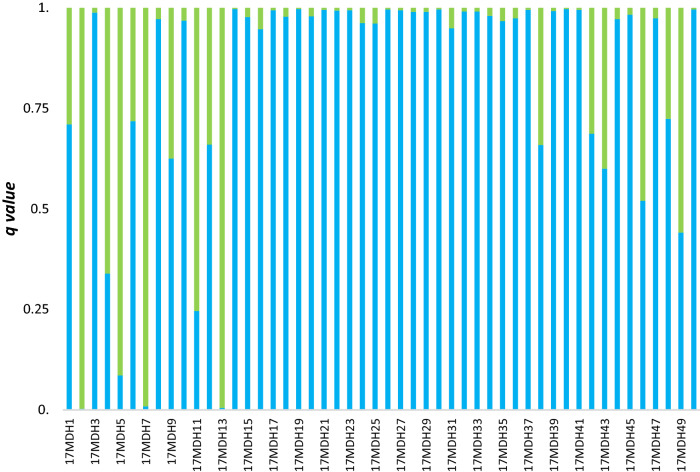
Average membership coefficient *q* from five independent runs for K = 2 calculated for 50 juveniles sampled in the potential hybridization zone in 2017. Green bars represent clade 1 (upstream of the barriers) and blue bars represent clade 2 (area of free migration).

**Figure 5:**
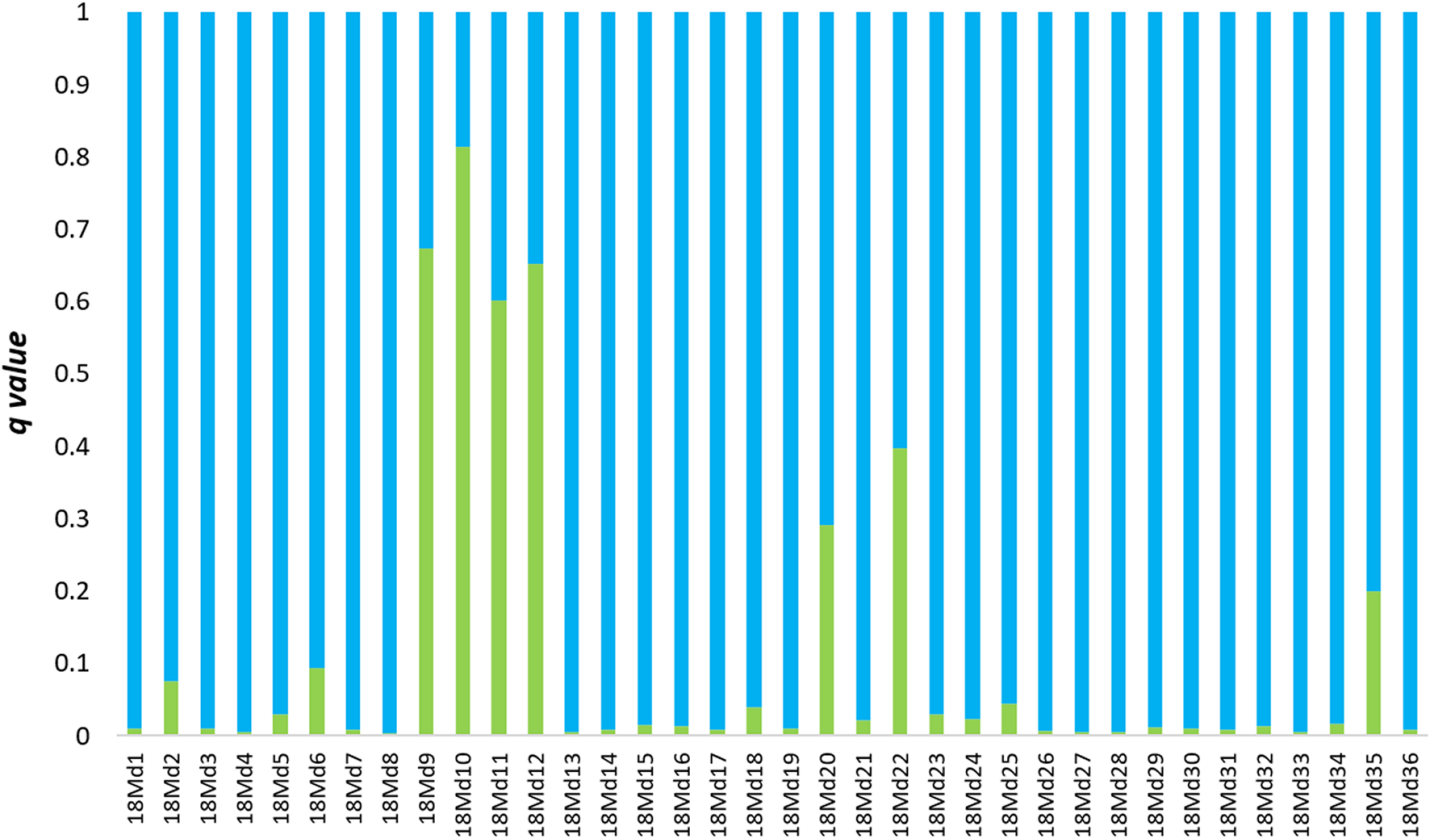
Average membership coefficient *q* from five independent runs for K = 2 calculated for 36 juveniles sampled in the potential hybridization zone in 2018. Green bars represent clade 1 (upstream of barriers) and blue bars represent clade 2 (area of free migration).

The results obtained from genetic structure analysis showed a potential hybridization zone located in the Mogilica River, and further calculations were done for this area. In other potential locations either migration was not completely blocked (Pokrzywnica and Gęsia rivers) or there were no trout upstream of the barriers (Wogra River). Juvenile trout from the hybridization zone in the Mogilica River were compared to trout upstream of the barrier and anadromous spawners from the same years. Hybrid detection based on the STRUCTURE membership coefficient showed the presence of potential hybrids in both 2017 and 2018 (Figs. 4, 5). The number of potential hybrids among juveniles (17MDH and 18MDH) was 12 in 2017 (24%) and 6 (16.6%) in 2018 at a threshold of q 0.2–0.8. Additionally, analysis based on samples from 17MDH indicated the presence 4 individuals (8%) that were F0 migrants from upstream of the barriers. In 18MDH, one upstream F0 migrant was found.

**Figure 6:**
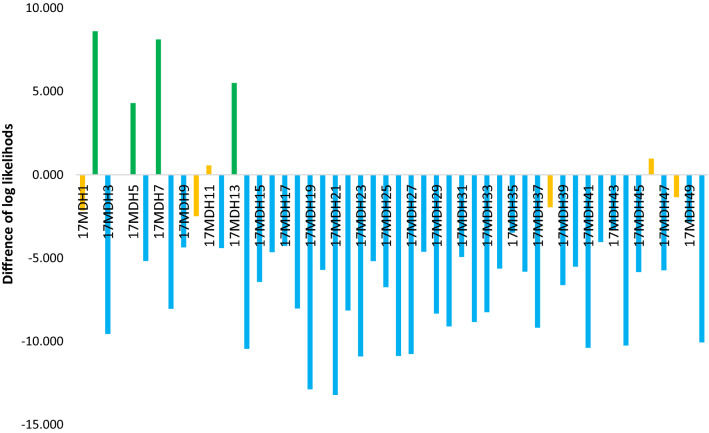
Results of the pairwise assignment test calculated as the difference between log likelihood of assignment to the 17MU and 17MDH locations for 50 juveniles from the hybridization zone in 2017. Specimens with negative values were more strongly related to the population downstream of the barrier. Green bars = F0 migrants, orange bars = hybrids, blue bars = anadromous genotypes.

**Figure 7:**
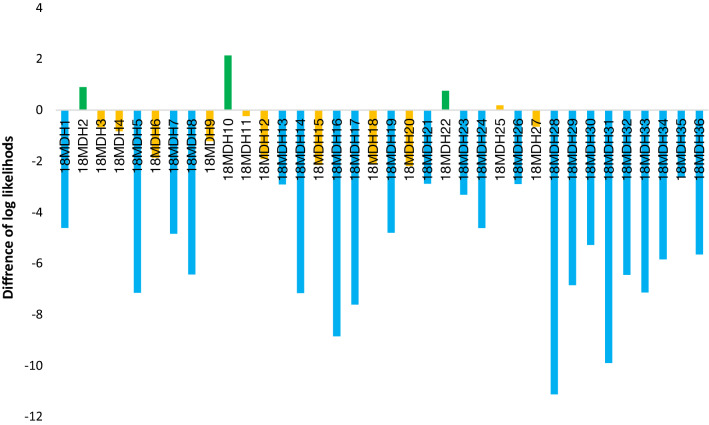
Results of the pairwise assignment test calculated as the difference between the log likelihood of assignment to 18MU and 18MDH locations for 36 juveniles from the hybridization zone in 2018. Specimens with negative values were more strongly related to the population downstream of the barrier. Green bars = F0 migrants, orange bars = hybrids, blue bars = anadromous genotypes.

The results obtained from the pairwise assignment tests in GenAlex 6.5 were generally congruent with Bayesian estimations, and, in most cases, the same fish were detected as hybrids or F0 upstream migrants. In the computations, the trout upstream of the barrier (17MU in 2017 and 18MU in 2018) were compared to juveniles from the potential hybridization zone (17MDH in 2017 and 18MDH in 2018). Among the juveniles from the 17MDH samples collected in 2017, 7 fish were marked as putative hybrids (14%) and 4 as F0 migrants (8%) upstream of the barrier (Fig. 6). These were the same fish detected as migrants from upstream with the structure algorithm. Among the juveniles collected in the hybridization zone (18MDH) in 2018, the pairwise assignment test also indicated 11 potential hybrids (27.7%) (Fig. 7); all of the hybrids detected with STRUCTURE were among them. The presence of F0 migrants was not confirmed. All the hybrid fish detected with the assignment test were also detected with the Bayesian method.

### SNP diversity and microarray hybrid detection

Basic statistics were calculated on the SNP genotype matrix limited to samples from five sites totalizing 144 trout (Table 4). The number of polymorphic loci (NPL) was higher in adult anadromous trout (17PAM) and juveniles from the hybridization zone sampled in 2017 (17MDH). The mean number of alleles varied between 1.662 and 1.996. The lowest values were found in samples originating upstream of the barriers in the Mogilica River (18MUU and 17MU) at 1.662 and 1.768, respectively. The observed heterozygosity was also the lowest in these locations (Table 4). The number of loci with significant departures from the HWE was low and observed only in the 17PAM and 17MDH samples. Population-specific F_IS_ was insignificant (p < 0.05) in all stocks demonstrating general panmixia across these sites.

**Table 4:** Genetic diversity for five brown trout stocks from the Parsęta River basin based on the SNP genotypes.

Abbreviation	N	NPL	MNA	H_O_	H_E_	DHWE	F_IS_
18MUU	13	2566	1.662	0.361	0.35	0	− 0.032
17MU	24	2978	1.768	0.33	0.329	0	0
17MDH	43	3846	1.995	0.332	0.347	2	0.045
18MDH	24	3678	1.949	0.372	0.359	0	− 0.038
17PAM	40	3852	1.996	0.343	0.351	2	0.023

*N* number of individuals, *NPL* number of polymorphic loci, *MNA* mean number of alleles, *H*_*O*_ observed heterozygosity, *H*_*E*_ expected heterozygosity, *DHWE* loci deviating from the HWE after Bonferroni correction and population-specific F_IS_ (insignificant: p < 0.05 for the whole sampling).

**Figure 8:**
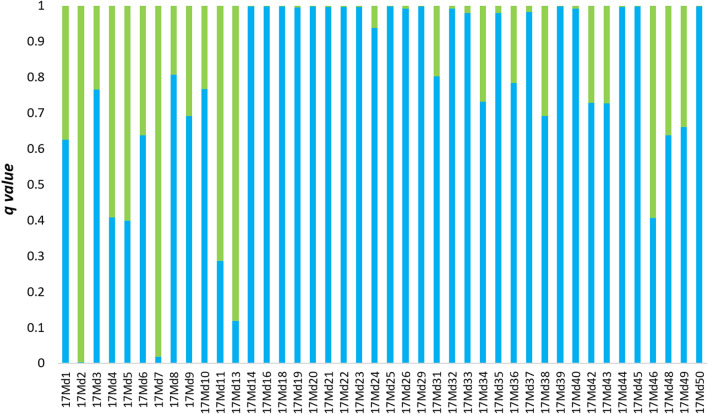
Average membership coefficient *q* from five independent runs for K = 2 calculated for 43 juveniles sampled in the potential hybridization zone in 2017 and genotyped with an SNP microarray. Green bars represent clade 1 (upstream of barriers) and blue bars represent clade 2 (area of free migration).

**Figure 9:**
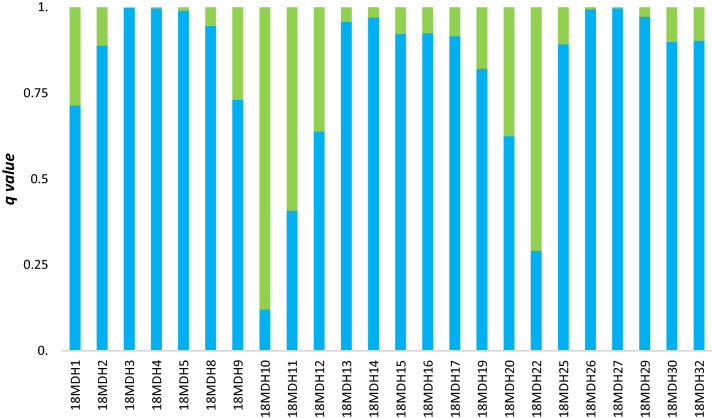
Average membership coefficient *q* from five independent runs for K = 2 calculated for 24 juveniles sampled in the potential hybridization zone in 2018 and genotyped with an SNP microarray. Green bars represent clade 1 (upstream of barriers) and blue bars represent clade 2 (area of free migration).

The overall F_ST_ for SNP loci obtained with molecular variance analysis was 0.069 and significant (AMOVA p < 0.05). The highest percentage of variation was detected within individuals at 91.75%. Overall F_IS_ and F_IT_ were 0.013 and 0.082, respectively, and were significant (P < 0.05). The value of general F_ST_ was caused by the distinctness of the samples collected upstream of the barriers (18MUU and 17MU). The dissimilarity of these samples was well evidenced by F_ST_ pairwise comparisons (Table S1). All the tests were significant (p < 0.05). The highest values of pairwise F_ST_ were obtained for the 17PAM vs. 18MC and 18MC vs. 18MDH comparisons. Next, Bayesian assignment analysis of genetic structure was performed using STRUCTURE 2.3.4. for the 144 brown trout genotyped on the microarray. The analysis confirmed that the maximum value of ΔK was K = 2 (ΔK = 195.5) (Supplementary Fig. S3). Brown trout from the tributary blocked by barriers comprised one cluster (17MU and 18MUU) and anadromous spawners (17PAM) with juveniles from the hybridization zone (17MDH and 18MDH) comprised the second cluster (Fig. S4). After computing the genetic structure, hybrid detection analysis was performed. Hybrid detection based on the STRUCTURE membership coefficient (0.8–0.2 threshold) indicated the presence of potential hybrids in both 2017 and 2018 (Figs. 8, 9). The number of potential hybrids among juveniles (17MDH and 18MDH) was 16 in 2017 (37%) and 7 in 2018 (29.6%). Additionally, analysis based on samples from 2017 indicated the presence of 3 migrants (F0) (6.9%) upstream of the barriers. In 2018, one F0 migrant was found.

## Discussion

The current study provided evidence that unidirectional gene flow occurred and that spawning grounds located downstream of impassable barriers could be hybridization zones between facultative anadromous and resident brown trout inhabiting upstream sections of rivers to which migration was blocked. Furthermore, this phenomenon occurred despite the large genetic distance between the downstream population in the free migration area and the upstream one in the blocked tributary. The results obtained from the 2 years studied showed not only the presence of hybrids but also recent migrants from upstream that could signal that some specimens from above the barrier either migrated or were moved downstream, e.g., with currents or high waters. The following discussion focuses on the genetic diversity of brown trout in the Parsęta River basin, the reliability of hybrid estimations, and the impact hybrids have had on the genetic structure of the population.

### Genetic diversity and genetic structure

The overall level of genetic diversity of the brown trout from the Parsęta River basin was moderate. The results for both molecular markers were similar. The genetic differentiation between populations situated upstream of barriers and those downstream of them in the rest of the basin was high or even very high (pairwise F_ST_ from 0.11 up to 0.216). On the other hand, the population from the rest of the basin was rather homogeneous probably because free migration was possible. The level of gene flow was illustrated well by the results of genetic structure analysis, which showed the extent of anadromous alleles. The overall F_ST_ was much lower (0.024) when only the free migration area was considered without juveniles from hybridization zones. This level of internal population differentiation was similar to that of neighboring Pomeranian rivers or the Vistula River^42,43^ and to that in anadromous populations from larger Baltic Sea rivers such as the Umealven, Laisalven, or Luga^44,45^. Evidence of genetic differentiation among brown trout populations that are fragmented by barriers are reported by many authors^46–48^; however, such high differences among neighboring locations in one river are reported less frequently^49^.

The genetic diversity calculated from microsatellite polymorphism data indicated very clearly that individuals from locations upstream of barriers had the lowest heterozygosity (mean Ho upstream = 0.51, downstream = 0.69), the lowest number of alleles, the lowest level of allelic richness and, almost no private alleles. In turn, the highest values of these parameters were noted in adult anadromous individuals. This tendency was also visible in the analysis with the SNP microarray, indicating that an isolated population had, typically, about 30% fewer polymorphic loci. Most likely, the loss of genetic diversity in isolated upstream zones resulted from genetic drift and probably the small population sizes in a small area of the basin of about 65 km^2^; this effect was similar to that of the founder effect. Considering that the barriers discussed date from the mid-nineteenth century, the high differentiation noted could have arisen over this period of time. An analysis of stocking history in the Parsęta River basin dating to the 1970s showed no evidence of stocking in this part of the basin and especially not upstream of barriers on the Mogilica River. Studies published recently on the genetic diversity of brown trout breeding lineages in Poland indicated that, despite high genetic diversity^43^, this isolated population was clustered together with breeding lineages from northern Poland.

### Level of hybridization, method reliability, and compatibility

The average number of hybrids in the hybridization zone was calculated at about 25% in each year. The number varied depending on the method, but the results obtained with SNP analysis were slightly higher than those based on microsatellites. This appeared to stem from differences in marker characteristics and because not all the individuals used in the microsatellite analysis were used in the SNP microarray analysis; insufficient DNA concentration in some samples from juveniles (due to in vivo sampling and small fish size) precluded their use in the SNP analysis. However, the compatibility of the two methods was confirmed as that (1) all the fish detected with microsatellite data as hybrids in the assignment test were also detected with the Bayesian method, and (2) all the hybrids detected from the microsatellite data were also detected in microarray analysis. In addition to the hybrids, the analysis also showed there were recent upstream migrants (F0) among the samples collected downstream of the barriers. This indicated that some individuals from upstream of the barriers swim (or are carried) down and cohabit with the progeny of anadromous fish. It is possible that some of them spawned with anadromous females as precocious males^50^. It would be interesting to know whether these individuals smoltify with the offspring of anadromous fish. The analysis of the genotypes of large, adult anadromous individuals in no way revealed the presence of a complete genotype that originated upstream of the barriers. However, taking into account their small numbers comparison with the entire population, this is not surprising. A recently published study^51^ indicated that in populations from the Imsa River in Norway that had been isolated by barriers for 25 years, the offspring of the resident form were less likely to choose an anadromous life strategy. This could indicate that, apart from the growth-density factor, genetic factors are also responsible for migration decisions. Hence, F0 migrants from upstream of the barriers probably preferred a resident strategy and did not migrate to the sea.

### Influence of hybrids on population genetic structure

The results of clustering indicated the consolidation of a small proportion of upstream genotypes among anadromous individuals. Thus, a certain level of gene flow occurred, which we would envisage as being a constant phenomenon. On the other hand, a certain proportion of anadromous genotypes was noted in individuals upstream of the first barrier (17MU, 18MU), but they were absent upstream of the next barrier (18MUU). So what was the effect of the exchange of the gene pool with the isolated upstream population on the anadromous population? The analysis indicated that this exchange did not increase variability in the whole population, but indeed, it probably reduced it. Among individuals from the isolated part of the river basin, lower rates of polymorphism were found with a simultaneous lack of private alleles. A study from Denmark^48^ on the effects of medieval dams on genetic divergence and the demographic history of brown trout populations in the Gudenå River suggested that the most important consequence of the dams was local adaptation and evolutionary potential where barriers imposed strong selection against anadromy. The results obtained in this study indicated a similar impact, and the isolated upstream part of the population became a reservoir of forced residency.

## Conclusions

Several conclusions can be drawn based on the results of this study. The brown trout population from the Parsęta River was slightly diversified internally in the area that was accessible to migration. At the same time, the isolated upstream part of the population was very different from that in the rest of the basin. The spawning areas of the anadromous form located downstream of the barriers were in a hybridization zone and unidirectional gene flow was observed. A few juveniles from blocked tributaries migrated downstream and shared nursery areas near anadromous spawning grounds, and genotypes typical of populations upstream of the barrier were admixed downstream in the population, but they constituted a small percentage. The lack of genotypes noted upstream of the barriers among anadromous adult individuals could have indicated that migrants of upstream origin and hybrids preferred residency.

## Supplementary Information

Supplementary Information 1.

Supplementary Information 2.

Supplementary Information 3.

Supplementary Information 4.

Supplementary Information 5.

Supplementary Information 6.

Supplementary Information 7.

Supplementary Information 8.

## Data Availability

The datasets used and analysed during the current study are available as Supplementary files Table S1 and Table S2.
