# Supplementary material for: Evidence of unidirectional gene flow in a fragmented population of Salmo trutta L
Source: Sci Rep. 2021 Dec 3;11:23417. doi: 10.1038/s41598-021-02975-9 (PMC8642411; doi:10.1038/s41598-021-02975-9)
Supplement: Supplementary file 1 — Supplementary Information 1. [file 41598_2021_2975_MOESM1_ESM.docx]

Descriptions for Supplementary files

Fig. S1. Plot from Evanno method. Highest value of (ΔK = 125.4) on K = 2

Fig. S2. Plot from Evanno method. Highest value of (ΔK 493.65) on K = 2

Fig. S3. Plot from Evanno method. Highest value of (ΔK = 195.5) on K = 2

Fig. S4. Clustering of 144 brown trout genotyped on an SNP microarray with putative K = 2.

Table S1. F_ST_ values for pairwise comparisons of five brown trout stocks from the Parsęta River basin calculated with *Salmo trutta* 5K SNP microarray.

Table S2. Complex genotypes of 13 microsatellite loci for 579 individuals of sea trout from the Parsęta river basin

Table S3. Genotypes for 144 individuals of sea trout from the Parsęta river basin analyzed with *Salmo trutta* 5K SNP microarray
