# Supplementary figures and images for: Evidence of unidirectional gene flow in a fragmented population of Salmo trutta L
Source: Sci Rep. 2021 Dec 3;11:23417. doi: 10.1038/s41598-021-02975-9 (PMC8642411; doi:10.1038/s41598-021-02975-9)

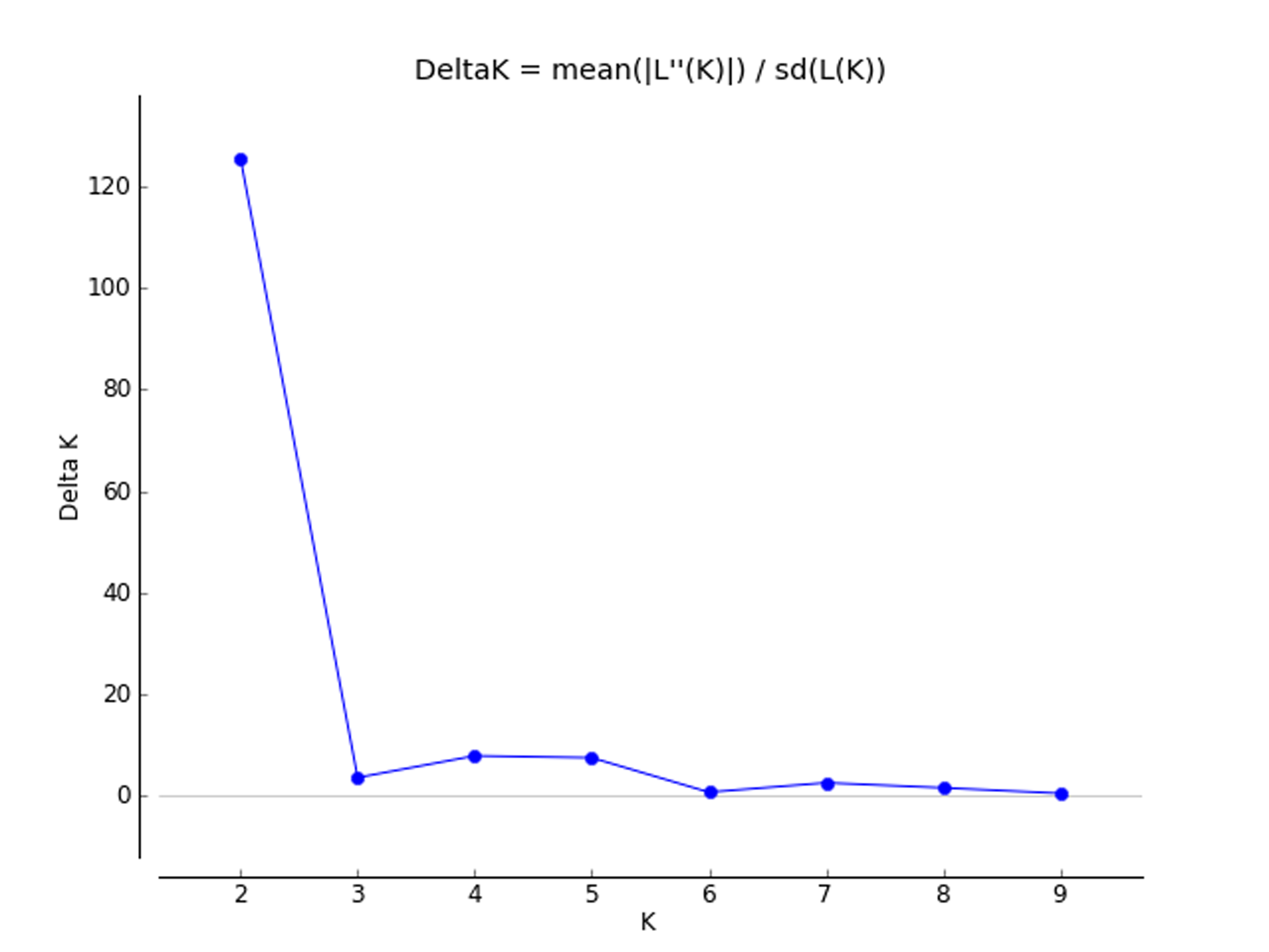

Supplement: Supplementary file 2 — Supplementary Information 2. [file 41598_2021_2975_MOESM2_ESM.tif]

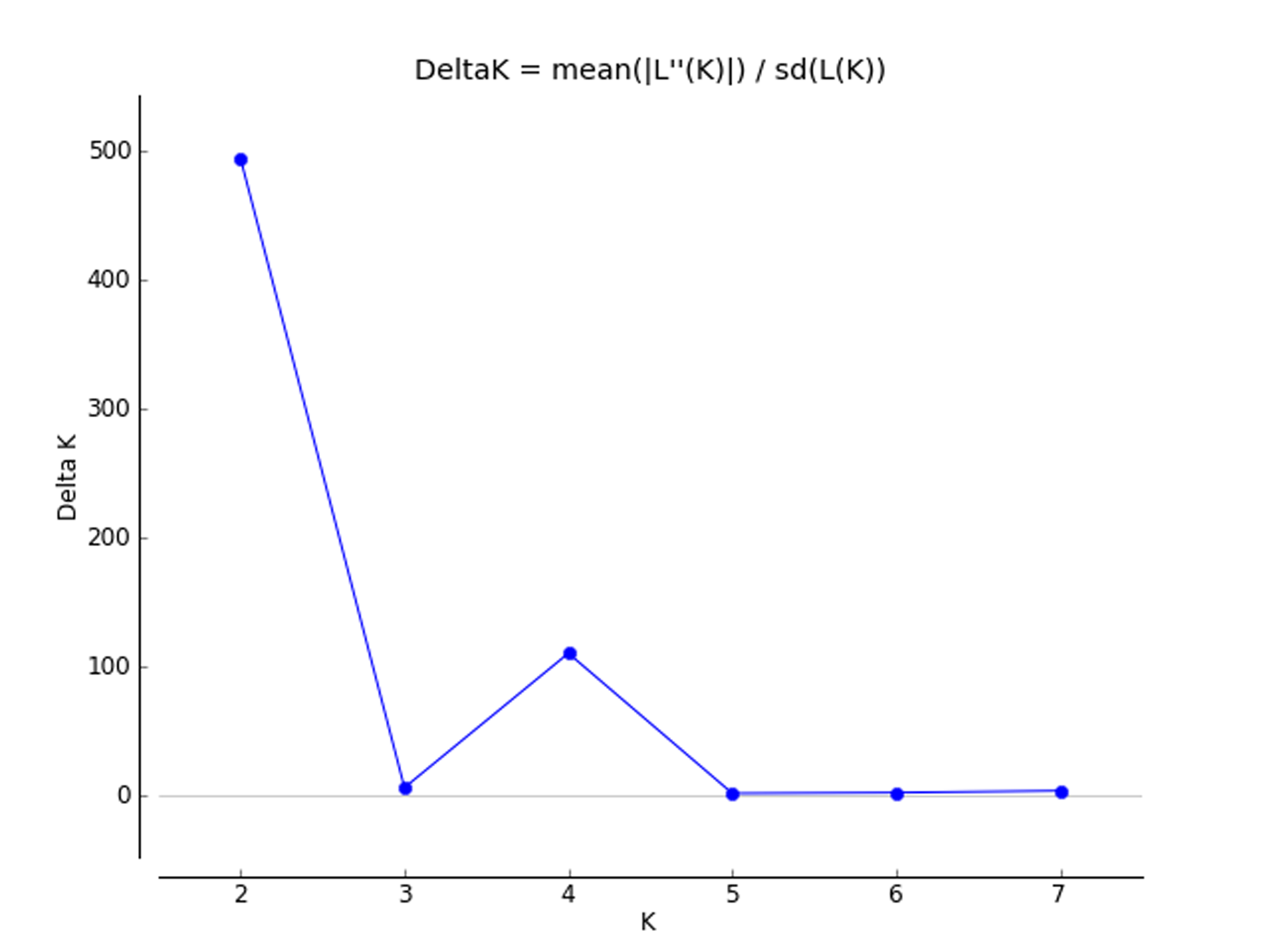

Supplement: Supplementary file 3 — Supplementary Information 3. [file 41598_2021_2975_MOESM3_ESM.tif]

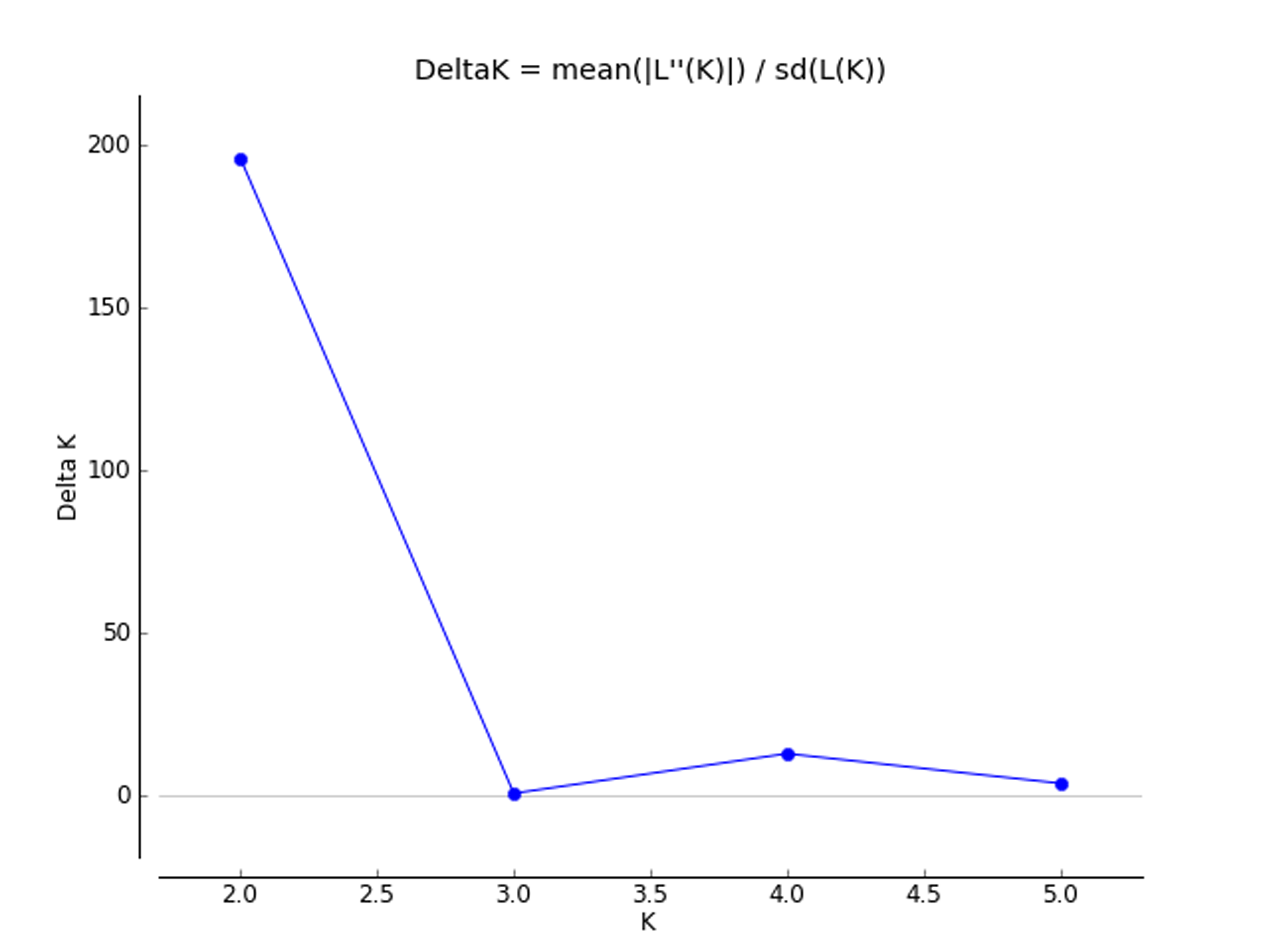

Supplement: Supplementary file 4 — Supplementary Information 4. [file 41598_2021_2975_MOESM4_ESM.tif]

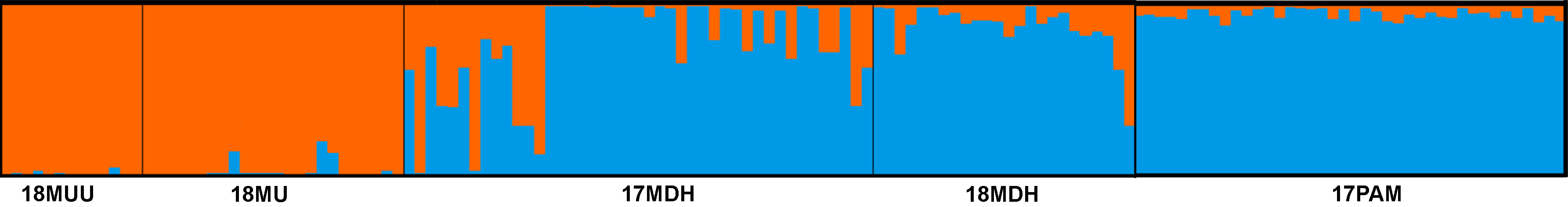

Supplement: Supplementary file 5 — Supplementary Information 5. [file 41598_2021_2975_MOESM5_ESM.tif]
