# Supplementary material for: Evidence of unidirectional gene flow in a fragmented population of Salmo trutta L
Source: Sci Rep. 2021 Dec 3;11:23417. doi: 10.1038/s41598-021-02975-9 (PMC8642411; doi:10.1038/s41598-021-02975-9)
Supplement: Supplementary file 6 — Supplementary Information 6. [file 41598_2021_2975_MOESM6_ESM.docx]

Table S1. Below the diagonal: F_ST_ values for pairwise comparisons of five brown trout stocks from the Parsęta River basin calculated with *Salmo trutta* 5K SNP microarray. All values were significant at p = 0.05; on the diagonal: the average number of pairwise differences within populations; and above the diagonal: Nei’s genetic distance D_A_

| **Abbreviation** | **18MUU** | **17MU** | **17MDH** | **18MDH** | **17PAM** |
| --- | --- | --- | --- | --- | --- |
| **18MUU** | 897.969 | 23.854 | 155.719 | 199.593 | 213.968 |
| **17MU** | 0.024 | 981.019 | 122.64 | 165.032 | 180.526 |
| **17MDH** | 0.108 | 0.091 | 1336.309 | 27.329 | 20.231 |
| **18MDH** | 0.143 | 0.125 | 0.02 | 1319.499 | 32.638 |
| **17PAM** | 0.143 | 0.129 | 0.015 | 0.024 | 1351.375 |
